# Targeting lysozyme 2 in remote endocardial zones promotes rapid cardiac repair after injury

**DOI:** 10.1186/s13619-025-00267-5

**Published:** 2025-11-13

**Authors:** Xiuxiu Liu, Bin Zhou

**Affiliations:** 1 CAS CEMCS-CUHK Joint Laboratory, New Cornerstone Science Laboratory, Key Laboratory of Multi-Cell Systems, Shanghai Institute of Biochemistry and Cell Biology, Center for Excellence in Molecular Cell Science, Chinese Academy of Sciences, University of Chinese Academy of Sciences, Shanghai, China; 2 Key Laboratory of Systems Health Science of Zhejiang Province, School of Life Science, Hangzhou Institute for Advanced Study, University of Chinese Academy of Sciences, Hangzhou, China; 3 School of Life Science and Technology, ShanghaiTech University, Shanghai, China

**Keywords:** Heart regeneration, Lysozyme 2, Cardiac injury, Infarct and remote zones, Endocardial

## Abstract

The adult mammalian heart lacks the capacity to regenerate after injury, leading to heart failure. While most of the research focused on the cardiomyocyte proliferation around the infarct zones, a new study (Fan et al., Cell Stem Cell 32(1563-1576):e1511, 2025) reveals a novel mechanism in the remote endocardial zone. They identified lysozyme 2 (Lyz2) as a critical regulator, where its sustained activity in the non-regenerative hearts promotes lysosomal degradation of the extracellular matrix (ECM). Then, the breakdown of ECM was found to induce cardiomyocyte apoptosis near the endocardium. Importantly, both the genetic deletion of *Lyz2* or the pharmacological inhibition of lysosomal degradation activity in mice after myocardial infarction (MI) preserved the ECM, reduced cardiomyocyte apoptosis, diminished scarring, and improved cardiac function. This work highlights LYZ2 as a novel therapeutic target for promoting heart repair in humans.

## Main text

Heart failure remains a leading cause of death worldwide, largely due to the inability of the adult mammalian heart to regenerate after injury. Unlike neonatal hearts, which can fully regenerate after apical resection (Bergmann et al. 2009), the adult heart responds to ischemic insults such as MI with fibrotic scarring and pathological remodeling. Current therapeutic strategies or fundamental research have focused primarily on enhancing cardiomyocyte proliferation (Mohamed et al. 2018) or promoting coronary angiogenesis (Das et al. 2019) within the infarct zone. Studies have shown that collateral arteries can help restore blood flow (Das et al. 2019). Additionally, cardiomyocytes located in the border zones of infarction exhibit higher cell cycle activity compared to those in remote zones (Senyo et al. 2013). However, a new study by Fan et al., recently published in *Cell Stem Cell*, shifts the paradigm by identifying *Lyz2* as a key regulator of cardiac repair in remote endocardial zones, which are far away from the initial injury site. This work not only reveals a previously unexplored mechanism of heart regeneration but also proposes a novel therapeutic strategy with translational potential.

While neonatal mouse hearts can fully regenerate following apex resection (Enzo R. Porrello 2011), this capacity diminishes rapidly after birth and is largely absent in adults (Pu et al. 2022). The sharp decline in regenerative potential around postnatal day 7 (P7) marks a critical transition from a regenerative to a non-regenerative state, making it a key focus of research. Previous works have compared transcriptomes of regenerative neonatal hearts and non-regenerative adult hearts to uncover the underlying regulatory mechanisms. These investigations revealed that inhibition of fatty acid oxidation can enhance heart regeneration (Ajit Magadum 2020; Li et al. 2023). Using spatial transcriptome and single-cell RNA sequencing, Fan et al. compared regenerative (postnatal day 1, P1) and non-regenerative (P7) mouse hearts after apical resection (Fan et al. 2025). They identified a transient but significant upregulation of *Lyz2* in both the infarct zones and remote left ventricle endocardial zones (LVEZs) of the regenerative hearts. In contrast, the *Lyz2* expression persisted in non-regenerative hearts, suggesting that sustained *Lyz2* activity may impede repair. Immunofluorescent staining confirmed that LYZ2 was specifically restricted in the endocardial cells within the LVEZs, but not in the infiltrating immune cells, highlighting a non-canonical role for *Lyz2* beyond its classical function as a myeloid cell marker. Further analysis by the transcriptome analysis revealed that *Lyz2* expression was associated with genes involved in lysosomal degradation (e.g., CTSB) and ECM remodeling. Pseudotime trajectory analysis revealed that *Lyz2* expression peaked between 2 to 4 days post-injury in the regenerative hearts, coinciding with the activation of endocardial cell markers and ventricular remodeling genes. This spatial–temporal pattern suggests that *Lyz2* is part of the coordinated response after cardiac injury.

To functionally characterize *LYZ2*, identified by the transcriptome analysis after cardiac injury, Fan et al. turned to human iPSC-derived endocardial-like cells (hEnLCs). Overexpression of LYZ2 enhanced the lysosomal activity, evidenced by DQ-BSA signal-staining and lysotracker signals. On the contrary, knocking down *LYZ2* reversed injury-induced heparan sulfate proteoglycan degradation (HSPG), a key component of the ECM enriched in the endocardium. These findings revealed LYZ2 as a positive regulator of lysosomal degradation capacity in hEnLCs, promoting ECM breakdown in response to injury. Notably, this lysosomal degradation activity was independent of TFEB, a master regulator of lysosomal biogenesis, indicating a distinct mechanism related to the LYZ2 resistance in the hEnLCs. The authors further demonstrated that LYZ2 induction in hEnLCs was triggered by soluble factors released from damaged myocardium, reinforcing the concept of a remote injury signal. In a co-culture system mimicking the human endomyocardium using iPSC-derived hEnLCs and hiCMs, knockdown of *LYZ* in hEnLCs led to increased HSPG levels and reduced apoptosis in adjacent cardiomyocytes. The anti-apoptotic effect underscores the importance of ECM preservation in maintaining cardiomyocyte viability. In addition to the established role of the extracellular matrix components like Agrin in promoting cardiomyocyte proliferation and heart regeneration via Dag1 inhibition (Bassat et al 2017; Morikawa et al 2017), Fan et al. provided another benefit of ECM: it exerts an anti-apoptotic effect on nearby cardiomyocytes.

These in vitro findings were validated in vivo using a constitutive *Lyz2* knockout mouse model. Following MI, *Lyz2* KO mice exhibited increased HSPG levels in the LVEZs, reduced cardiomyocyte apoptosis, and larger preserved endocardial zones compared to wild-type controls. Importantly, these *Lyz2* KO mice showed significantly improved systolic function within one week post-MI, with enhanced ejection fraction and reduced fibrosis sustained for 28 days. As these benefits were observed in both adult and P7 injury models, the therapeutic relevance of *Lyz2* deletion across developmental stages is confirmed. A future investigation using an endocardial-specific *Lyz2* knockout could determine if the protective effect is locally mediated.

Given the promising genetic results, the authors explored pharmacological inhibition of lysosomal degradation as a potential therapeutic strategy. In hEnLCs, the co-administration of CA-074Me (a lysosomal inhibitor) and apilimod (a PIKFYVE inhibitor) increased HSPG retention and reduced apoptosis in co-cultured cardiomyocytes. Subsequent in vivo validation confirmed this inhibitor regimen, administered after MI, mirrored the effects of the *Lyz2* KO model: it enhanced HSPG levels, reduced apoptosis, improved cardiac function, and diminished scar formation. These benefits were evident within one week and sustained for 28 days. Importantly, apilimod is already in clinical trials for other indications, a factor that could accelerate its repurposing for cardiac repair. The pharmacological approach provides a viable alternative to genetic interventions and underscores the translational potential of targeting lysosomal degradation.

## Conclusions

This work redefines *Lyz2* as a remote-injury-triggered regulator of lysosomal degradation that exacerbates ECM breakdown and impedes cardiac repair. By shifting the focus from the infarct zone to remote endocardial regions, Fan et al. provide compelling evidence that targeting *Lyz2*—genetically or pharmacologically, preserves ECM integrity, reduces cardiomyocyte apoptosis in remote zones, and thereby promotes rapid functional recovery after MI. This study not only expands our understanding of cardiac repair beyond cardiomyocytes and local infarct but also identifies a readily translatable intervention strategy. Furthermore, it raises intriguing questions about inter-tissue communication, proposing ERK/STAT3 signaling as a potential mediator of this remote response. Future work to elucidate how injury signals propagate, why *Lyz2* is downregulated in regenerative hearts, and how to optimize delivery methods for clinical application could offer new hope for patients with ischemic heart disease.

## Data Availability

Not applicable.

## Abbreviations

Lyz2: Lysozyme 2
ECM: Extracellular matrix
MI: Myocardial infarction
P7: Postnatal day 7
LVEZs: Left ventricle endocardial zones
hEnLCs: Human iPSC-derived endocardial-like cells
HSPG: Heparan sulfate proteoglycan degradation
